# Improving the synergistic combination of programmed death‐1/programmed death ligand‐1 blockade and radiotherapy by targeting the hypoxic tumour microenvironment

**DOI:** 10.1111/1754-9485.13416

**Published:** 2022-04-24

**Authors:** Faiqa Mudassar, Han Shen, Kristina M Cook, Eric Hau

**Affiliations:** ^1^ Translational Radiation Biology and Oncology Laboratory, Centre for Cancer Research The Westmead Institute for Medical Research Sydney New South Wales Australia; ^2^ Sydney Medical School The University of Sydney Sydney New South Wales Australia; ^3^ Charles Perkins Centre The University of Sydney Sydney New South Wales Australia; ^4^ Department of Radiation Oncology, Crown Princess Mary Cancer Centre Westmead Hospital Sydney New South Wales Australia; ^5^ Blacktown Hematology and Cancer Centre Blacktown Hospital Sydney New South Wales Australia

**Keywords:** hypoxia, immune suppression, PD‐1/PD‐L1 blockade, radiotherapy, tumour microenvironment

## Abstract

Immune checkpoint inhibition with PD‐1/PD‐L1 blockade is a promising area in the field of anti‐cancer therapy. Although clinical data have revealed success of PD‐1/PD‐L1 blockade as monotherapy or in combination with CTLA‐4 or chemotherapy, the combination with radiotherapy could further boost anti‐tumour immunity and enhance clinical outcomes due to the immunostimulatory effects of radiation. However, the synergistic combination of PD‐1/PD‐L1 blockade and radiotherapy can be challenged by the complex nature of the tumour microenvironment (TME), including the presence of tumour hypoxia. Hypoxia is a major barrier to the effectiveness of both radiotherapy and PD‐1/PD‐L1 blockade immunotherapy. Thus, targeting the hypoxic TME is an attractive strategy to enhance the efficacy of the combination. Addition of compounds that directly or indirectly reduce hypoxia, to the combination of PD‐1/PD‐L1 inhibitors and radiotherapy may optimize the success of the combination and improve therapeutic outcomes. In this review, we will discuss the synergistic combination of PD‐1/PD‐L1 blockade and radiotherapy and highlight the role of hypoxic TME in impeding the success of both therapies. In addition, we will address the potential approaches for targeting tumour hypoxia and how exploiting these strategies could benefit the combination of PD‐1/PD‐L1 blockade and radiotherapy.

## Background

Immunotherapy is a validated cancer therapy that functions to activate the body's natural immune response against cancer cells.^1^ Over the past decade, there have been significant clinical advances in cancer immunotherapy, especially in the field of immune checkpoint inhibition (ICI).^2^ Immune checkpoints are receptor‐ligand pairs that regulate T‐cell activation at various stages of an immune response and help prevent autoimmunity.^3^ The two most studied immune checkpoint receptors that are expressed on the T cells include cytotoxic T lymphocyte‐associated antigen‐4 (CTLA‐4) and programmed death‐1 (PD‐1). CTLA‐4 competes with a costimulatory molecule CD28, also expressed on the T cells, and both these molecules bind to CD80 and CD86 on antigen‐presenting cells (APCs). In contrast, PD‐1 binds to its ligands, programmed death ligand‐1 (PD‐L1) and programmed death‐ligand 2 (PD‐L2), which are also present on APCs. The binding of these checkpoint receptors to their respective ligands inhibits T‐cell activation and proliferation, thus dampening the immune response.^4^, ^5^

In recent years, PD‐1/PD‐L1 blockade has shown promise in clinical trials and is at the forefront of cancer immunotherapy.^6^ It has been trialled in various solid malignancies, resulting in significant anti‐tumour effects including in melanoma, renal cell carcinoma, non‐small cell lung cancer (NSCLC), colorectal cancer, urothelial carcinoma, head and neck carcinomas, and hepatocellular carcinoma (HCC).^7^, ^8^, ^9^, ^10^, ^11^, ^12^, ^13^, ^14^ However, despite long‐term remission and potentially cure‐like benefits, inherent resistance to PD‐1/PD‐L1 blockade monotherapy remains a clinical challenge. Additionally, most responders may experience acquired resistance and eventually develop clinical relapse after a few years.^15^, ^16^

The tumour microenvironment (TME) has a major role in determining tumour immunogenicity and resistance to PD‐1/PD‐L1 blockade.^17^ Tumours are normally divided into immunogenic (or hot) and poorly immunogenic (or cold) tumour types. A hot TME is infiltrated with more immune cells, for example effector T cells, natural killer (NK) cells and dendritic cells (DCs), compared with a cold TME with low‐to‐no immune cell infiltration. Tumours with an immunogenic phenotype have improved prognosis and can respond well to ICI.^18^, ^19^, ^20^ Moreover, the presence of immunosuppressive cells such as regulatory T cells (Tregs), myeloid‐derived suppressor cells (MDSCs) and anti‐inflammatory macrophages in the TME also plays an important role in resistance to anti‐PD‐1/PD‐L1 therapy.^17^ This suggests that modifying the TME to create an immunogenic phenotype and limit the recruitment and function of immunosuppressive cells could enhance the efficacy of PD‐1/PD‐L1 blockade therapy. Therefore, combination of anti‐PD‐1/PD‐L1 antibodies with various other immunostimulatory therapies that target the TME, including radiotherapy, is actively being investigated.^4^ The combination of PD‐1/PD‐L1 blockade with radiotherapy can boost the anti‐tumour responses and result in better clinical outcomes.

It is also important to understand that the success of the combination approach can be compromised by additional TME‐associated factors such as tumour hypoxia. Hypoxia, a common feature of most solid tumours, generates an immunosuppressive TME, thereby suppressing the anti‐tumour immune responses.^21^, ^22^ Hence, overcoming the immunosuppressive mechanisms driven by the hypoxic TME may be an effective approach to elicit durable anti‐tumour immunity and improve the responses of combined PD‐1/PD‐L1 blockade and radiotherapy. In this review, we will provide a brief overview of the combination of PD‐1/PD‐L1 blockade with radiotherapy. Furthermore, we will discuss how tumour hypoxia can act as a barrier to blunt the efficacy of both therapies. Finally, we will address how targeting the hypoxic TME has the potential to improve the synergistic interaction of PD‐1/PD‐L1 blockade and radiotherapy.

## The PD‐1 pathway

As a member of the B7/CD28 family of costimulatory T‐cell receptors, PD‐1 is upregulated upon T‐cell activation and during persistent antigen encounter, such as in the cases of chronic infections and cancers. Along with activated T cells, PD‐1 is also widely expressed by Tregs, B cells, myeloid cells, DCs, NK cells and tumour‐infiltrating lymphocytes (TILs) from different tumour types.^3^ Its ligand PD‐L1 is present on T cells, B cells, DCs, macrophages and other non‐immune cells.^23^ PD‐L1 is also found to be upregulated on several solid tumours and is linked to increased TILs and poor patient prognosis.^24^, ^25^, ^26^ In contrast, the expression of PD‐L2 is limited, and it is mainly expressed on DCs and activated macrophages.^27^

Under normal conditions, the PD‐1/PD‐L1 pathway plays an essential role in maintaining immune tolerance and preventing autoimmune diseases.^4^ PD‐1/PD‐L1 inhibit T‐cell responses and suppress overstimulation of the immune response, essentially acting as a break or an off‐switch for the immune system. Tumours can exploit this pathway by overexpressing PD‐L1 which impairs anti‐tumour immunity and inhibits the activation and function of T cells by decreasing the production of inflammatory cytokines while increasing the secretion of inhibitory cytokines, decreasing lymphocyte proliferation and cytotoxicity, and stagnating the T‐cell cycle.^4^, ^28^, ^29^ The increased expression of PD‐1 is also a hallmark of T‐cell exhaustion, resulting in T‐cell dysfunction and lack of immune surveillance in infections and tumours.^30^, ^31^ Thus, blocking the PD‐1/PD‐L1 pathway can preserve the anti‐tumour capacity of T cells and prevent immune evasion of the tumour cells.

## Radiotherapy

Radiotherapy remains a key component of cancer treatment, with curative or palliative roles in different cancer types. It uses ionizing radiation to directly damage the DNA by inducing DNA breaks. Indirect effects of radiation are mediated via reactive oxygen species (ROS) that oxidize lipids and proteins, and these also induce damage in the DNA. The consequence of radiation‐induced DNA damage is programmed cancer cell death and mitotic failure.^32^ Unfortunately, many cancers are either inherently radiation resistant or they can develop acquired radioresistance following radiotherapy. Thus, the effectiveness of radiotherapy is frequently improved by combination with surgery, chemotherapy and immunotherapy.

## Combination of PD‐1/PD‐L1 blockade and radiotherapy

### Influence of radiation on the immune system

**Fig. 1:**
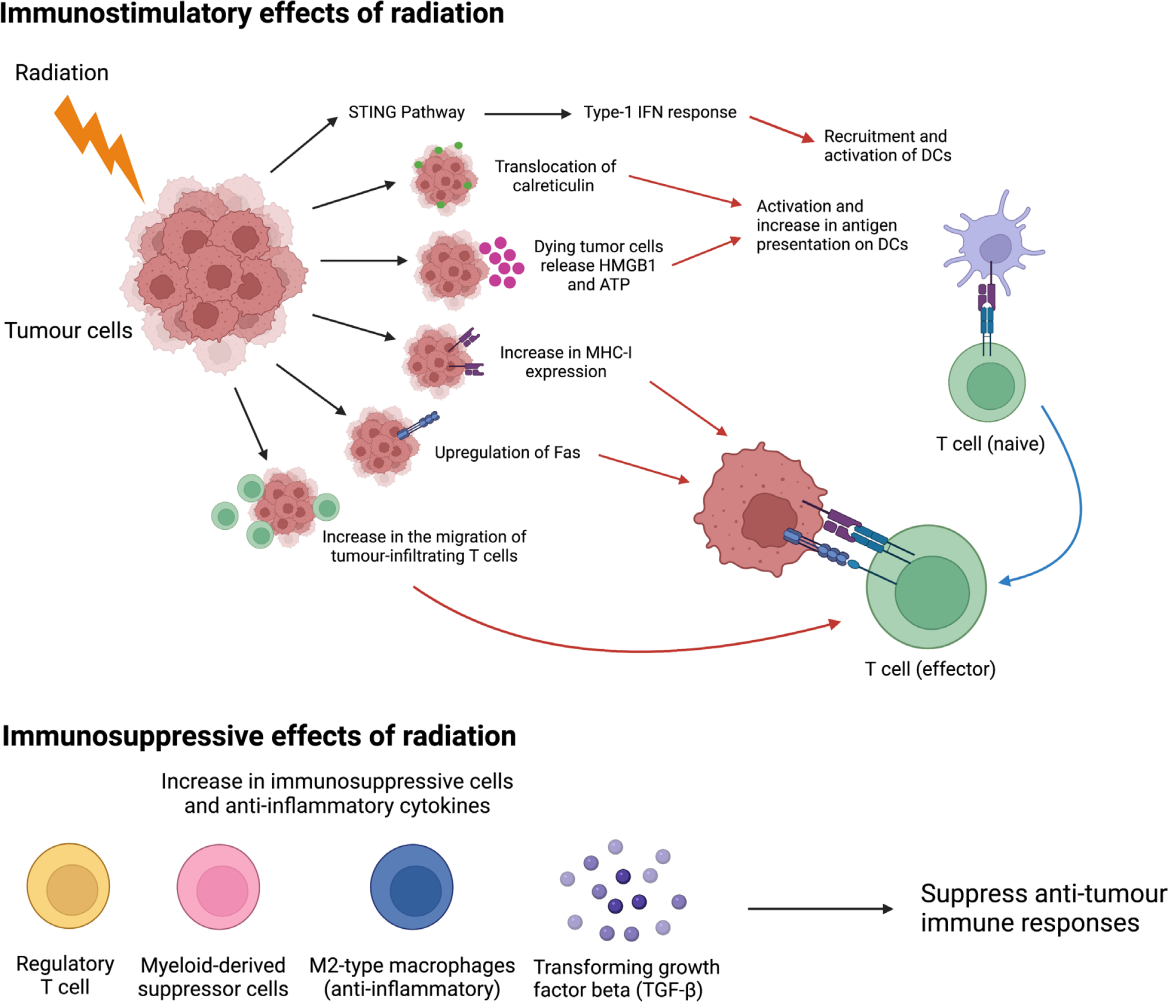
Radiation can stimulate the immune response and induce immunogenic cell death in several ways. It activates the stimulator of interferon genes (STING) pathway that induce type‐1 interferon (IFN) response which aids in the recruitment and activation of dendritic cells (DCs). Radiation can induce various damageassociated molecular pattern (DAMP) molecules including calreticulin, high‐mobility group box 1 (HMGB1) and adenosine triphosphate (ATP). All these DAMPs promote activation of DCs and increase antigenpresentation to the naïve T cells, which then convert to effector T cells. Radiation also increases the expression of major histocompatibility complex class I (MHC‐I) that present antigens to T cells, and Fas death receptor that can induce apoptotic cell death of the tumour cells. Radiation increases the migration of tumour‐infiltrating T cells to the tumour region that is required for tumour recognition and anti‐tumour effect. The immunosuppressive effects of radiation are mediated by radiation‐induced increase in the recruitment of immunosuppressive cells such as regulatory T cells, myeloid‐derived suppressor cells and M2‐type macrophages, and increase in the generation of anti‐inflammatory cytokines including transforming growth factor‐beta (TGF‐β). The immunosuppressive cells and cytokines can impair anti‐tumour immune responses, thus sustaining immunosuppression in the tumour microenvironment. (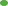) Calreticulin, (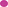) HMGB1 and ATP, (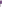) MHC‐I, (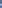) Fas death receptor, (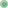) T cell, (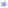) dendritic cell (DC), (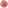) tumour cell, (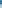) T cell receptor. Figure created using Biorender.com. [Colour figure can be viewed at wileyonlinelibrary.com]

Radiation acts as an immune system modulator as it can influence the immune state of the tumour in various ways (Fig. 1). Radiotherapy has been shown to promote tumour‐specific immune responses both in animal models and humans.^33^, ^34^ Radiation causes activation of the stimulator of interferon genes (STING) pathway that induces interferon‐I production, which is essential for recruiting and activating DCs in the TME.^35^ Radiation can induce various damage‐associated molecular pattern (DAMP) molecules that also activate the DCs and promote the uptake and presentation of tumour cell antigens by DCs. Calreticulin, an endoplasmic reticulum protein, is a DAMP molecule that serves as a signal for DCs to phagocytose the dying tumour cells.^36^ Other DAMPS including high‐mobility group box 1 (HMGB1) promote antigen cross‐priming on the T cells, and adenosine triphosphate (ATP) which activates inflammasome signalling in DCs, inducing IL‐1β that primes the T cells.^37^, ^38^ Chronic exposure of these DAMPs in the TME stimulates long‐lasting anti‐tumour immunity and results in immunogenic cell death. Additionally, irradiated tumour cells can upregulate the expression of major histocompatibility complex class I (MHC‐I) and cell surface death receptor Fas. Increased MHC‐I enhances recognition of irradiated tumour cell antigens by effector T cells, and upregulated Fas promotes apoptotic cell death.^39^, ^40^, ^41^ Moreover, radiation also enhances the trafficking and homing of tumour‐specific T cells to the tumour region, which is necessary for the anti‐tumour effect.^42^ Collectively, these immunostimulatory properties of radiation suggest that it can be exploited to improve the efficacy and overcome resistance to PD‐1/PD‐1 blockade immunotherapy.

Along with the immunostimulatory effects, several immunosuppressive effects are also reported following exposure to radiotherapy (Fig. 1). Radiation increases the recruitment of MDSCs, Tregs and anti‐inflammatory macrophages, all of which help sustain immune suppression in the TME.^43^, ^44^, ^45^ Furthermore, radiation‐induced ROS increases the anti‐inflammatory cytokine *transforming growth factor‐beta* (TGF‐β), which inhibits the activation of DCs and antigen presentation to T cells.^46^, ^47^ The immunosuppressive effects mediated by radiotherapy need to be controlled as these may oppose the proinflammatory states induced by radiation.

Although radiation could enhance the immunogenicity of tumours, it is still unable to elicit a coordinated and effective immune response as a monotherapy. This is because single‐site radiotherapy rarely results in tumour regression in unirradiated sites (known as abscopal effect).^48^ Increasing evidence indicates that abscopal effects are immune‐mediated responses and their infrequency in clinical settings following radiotherapy alone is likely due to the counterbalance of immunostimulatory and immunosuppressive effects of radiotherapy.^49^, ^50^, ^51^ Given that PD‐1/PD‐L1 blockade immunotherapy can activate anti‐tumour immune responses, it is possible that radiotherapy combined with PD‐1/PD‐L1 blockade immunotherapy can amplify immune responses against tumours and therefore increase the occurrence of abscopal effects.

### Synergistic interaction of PD‐1/PD‐L1 blockade and radiotherapy

Preclinical studies have demonstrated improved anti‐tumour responses from the combination of PD‐1 or PD‐L1 blockade and radiotherapy. As an example, the combination of anti‐PD‐1 antibody and radiation showed synergistic improvement in survival and significantly decreased tumour volume in preclinical mice models of intracranial glioma, melanoma and breast cancer.^52^, ^53^ Other studies in mice have also reported better tumour control with the combination of anti‐PD‐L1 antibody with radiation compared to either treatment alone.^54^, ^55^ More importantly, abscopal effects were observed in the combined therapy group only.^54^ Furthermore, in a PD‐1 resistant lung cancer model, radiation increased MHC‐1 production and interferon‐beta (IFN‐β) levels and sensitized this model to PD‐1 blockade therapy, suggesting that radiation has the potential to overcome PD‐1 resistance.^56^

Based on these promising findings, many clinical trials have also confirmed the synergistic interaction of PD‐1/PD‐L1 blockade and radiotherapy.^57^, ^58^ In particular, the KEYNOTE‐001 (phase 1), PEMBRO‐RT (phase 2) and PACIFIC (phase 3) trials have all supported the feasibility and effectiveness of combined PD‐1/PD‐L1 inhibition and radiotherapy.^58^, ^59^, ^60^ More clinical trials assessing the synergy between the combination are currently ongoing (as indicated by searching these terms on the ClinicalTrials.gov website).

### Considerations for the combination approach

Several factors need to be considered to maximize the therapeutic effect and minimize the risk of toxicity from the combination of PD‐1/PD‐L1 blockade and radiotherapy. These include the sequence of application (concurrent or sequential), radiation dose and fractions, and safety of the combination treatment.^61^, ^62^ While preclinical investigations and some clinical trials have favoured the concurrent application sequence of PD‐1/PD‐L1 blockade and radiotherapy,^55^, ^63^, ^64^ other trials have supported the effectiveness of sequential radiotherapy combined with PD‐1/PD‐L1 inhibition.^59^, ^60^ Moreover, preclinical studies found that radiation delivered as single high dose of 20 Gy could impair tumour immunogenicity, whereas fractionated radiotherapy could stimulate anti‐tumour immunity and demonstrated effective abscopal responses when combined with anti‐CTLA‐4 antibody.^65^, ^66^ However, clinical data to clearly support a difference between single and multi‐fraction radiotherapy schedules in combination with immune checkpoint blockade is still lacking. Currently, there is no consensus on the most effective treatment schedule for the combination approach. More clinical studies are needed to justify the dosages and mode of the combination therapy for optimal therapeutic benefit.

Along with the above‐mentioned considerations, the immunosuppressive mechanisms of hypoxia in the TME can also negatively affect the outcomes of the combined therapy approach. Tumour hypoxia reduces the efficacy of both radiotherapy and immunotherapy when used as monotherapies; therefore, it is highly likely that the hypoxic TME could impede the success of the combination approach by suppressing the anti‐tumour responses. Thus, to fully realize the benefits of this combination, another strategy could involve the incorporation of drugs that target hypoxia and/or the downstream effects of hypoxia to amplify the synergistic interaction of PD‐1/PD‐L1 blockade and radiotherapy.

## Hypoxia as a barrier to radiotherapy and PD‐1/PD‐L1 blockade immunotherapy

### Establishment of the hypoxic tumour microenvironment

Hypoxia occurs when the oxygen pressure in the TME drops below normal for the surrounding tissue. It arises due to the high oxygen consumption and rapid proliferation of tumour cells, leading to a mismatch between oxygen supply and demand, and resulting in the development of hypoxic niches.^67^, ^68^ The hypoxic TME induces angiogenesis, promoting the growth of neovascular networks which are often disorganized and aberrant, which further enhances tumour hypoxia.^69^, ^70^ Hypoxia influences tumour biology in several ways including promoting tumour invasiveness and metastasis, altering tumour metabolism, suppressing apoptotic responses and generating an immunosuppressive TME.^22^, ^71^, ^72^, ^73^, ^74^

Hypoxia also stabilizes the transcription factors, hypoxia‐inducible factors (HIFs), which maintain tumour cell adaptation to the hypoxic TME. There are three isoforms of the oxygen regulated component of HIF, that is HIF‐1α, HIF‐2α and HIF‐3α, and the hypoxic responses in the TME are primarily regulated by HIF‐1α. HIF pathways are upregulated in many tumour types and increased expression of HIFs is correlated with poor patient prognosis.^75^, ^76^, ^77^, ^78^ HIF activity also promotes several other biological pathways that contribute to resistance to radiotherapy and immunotherapy, which will be discussed in the following sections.^79^, ^80^

### Hypoxia impairs radiation response

**Fig. 2:**
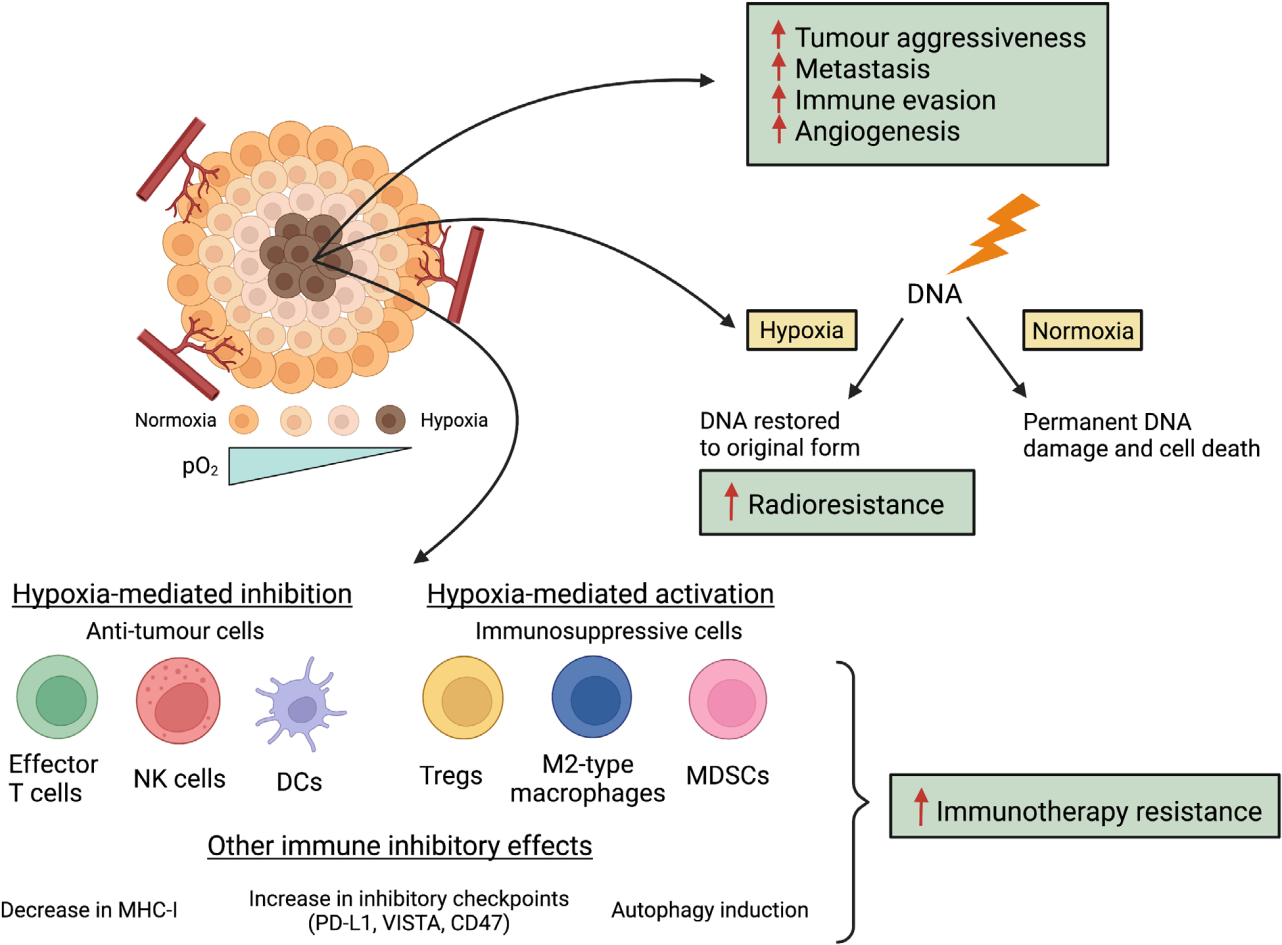
Hypoxia promotes tumour progression and leads to radioresistance and immunotherapy resistance. Hypoxia is a key player in the tumour aggressiveness and promotes metastasis, angiogenesis and tumour immune evasion. Hypoxia contributes to radioresistance by preventing permanent DNA damage from ionizing radiation and instead restoring the DNA back to its original form. However, under normoxic conditions, damaged DNA undergoes permanent fixation that eventually leads to cell death. Hypoxia also leads to immunotherapy resistance by modulating the tumour microenvironment (TME) in several ways. It inhibits the anti‐tumour immune cells such as effector T cells, natural killer (NK) cells and dendritic cells (DCs), while activating and increasing the recruitment of immunosuppressive cells which include regulatory T cells (Tregs), myeloid‐derived suppressor cells (MDSCs) and anti‐inflammatory M2‐type macrophages. Hypoxia can impair tumour‐antigen presentation to T cells by downregulating the expression of major histocompatibility complex class I (MHC‐I). It also increases various inhibitory checkpoint molecules such as programmed death‐ligand 1 (PD‐L1), V‐Domain Ig Suppressor of T‐cell Activation (VISTA) and cluster of differentiation 47 (CD47), all of which contribute to immune evasion. Moreover, hypoxia enhances autophagy, which can impair tumours susceptibility to immune cell attack such as inhibiting NK‐cell‐mediated killing of tumour cells. Figure created using Biorender.com. [Colour figure can be viewed at wileyonlinelibrary.com]

Hypoxia is a major barrier to the effectiveness of radiotherapy. It limits the efficacy of radiotherapy by attenuating the radiation‐induced DNA damage as oxygenation is essential for permanent DNA damage (Fig. 2).^81^, ^82^ Hypoxia confers a radioresistant phenotype as hypoxic tumours require a 2‐ to 3‐fold higher radiation dose than normoxic tumours to achieve the same biological effect.^83^ As hypoxia drives radioresistance of most cancers, alleviating tumour hypoxia is an attractive strategy to improve the radiosensitivity of tumours and enhance radiation outcomes.

### Hypoxia suppresses the anti‐tumour immune responses

The hypoxic TME strongly influences the immune cells and emerging evidence suggests that hypoxia is a key component in immunotherapy resistance (Fig. 2).^22^, ^84^, ^85^ Links between the hypoxic TME and the mechanisms of immunotherapy resistance are further supported by several preclinical and clinical studies.^86^ Hypoxia‐driven mechanisms, including those regulated by HIF, can result in immune tolerance and immune evasion by damaging the key regulatory anti‐tumour immune responses.^80^ Hypoxia also creates an immunosuppressive TME by recruiting immunosuppressive cells, upregulating inhibitory checkpoint proteins and altering the tumour metabolic landscape.^87^ Moreover, hypoxic regions of the tumours have poor infiltration of anti‐tumour immune cells and the limited immune cells that do reach the hypoxic areas are still unable to fully exhibit their anti‐tumour functions.^80^ In this section, we will provide a detailed overview of how hypoxia affects the different immune cell types and immune responses, with a particular focus on HIF‐1 regulated processes.

#### Effector T cells

Effector T cells, consisting of CD8+ cytotoxic and CD4+ helper T cells, are vital components of adaptive immune response against tumour antigens. Hypoxia alters the effector T‐cell population in several ways, including through inducing apoptosis, delaying the differentiation of T cells and reducing the release of proinflammatory cytokines such as interleukin‐2 and interferon‐gamma (IFN‐γ).^22^, ^88^ Hypoxia can impair the activation of T cells by decreasing the activation markers, CD40L and CD69.^89^ Furthermore, hypoxia via HIF‐1α pathway converts CD4+ T cells into the Tregs, which suppress the effector function of CD8+ T cells.^90^, ^91^ Hypoxia‐induced HIF‐1α enhances the accumulation of metabolic by‐product lactate, creating an acidic TME, which further suppresses T‐cell proliferation, cytokine production and inhibits their cytolytic activity.^92^, ^93^ Moreover, under hypoxic conditions, there is an increase in adenosine, an immunosuppressive metabolite that also hampers anti‐tumour immunity.^94^, ^95^ The binding of adenosine with its receptors (A2AR and A2BR) on the T cells, leads to the production of intracellular cyclic adenosine monophosphate (cAMP) which impair effector T‐cell functions such as T‐cell trafficking and the release of proinflammatory cytokines.^95^, ^96^

#### Dendritic cells

Dendritic cells are the dominant APCs that present tumour antigens to T cells and initiate immune responses. Hypoxia affects DCs by impairing their circulation and downregulating the expression of both differentiation and maturation markers (CD80, CD83 and MHC‐II), and costimulatory molecules (CD40, CD80 and CD86) on the DCs.^97^, ^98^ Hypoxia downregulates the chemokine receptor CCR7, which is required for mature DCs migration to the lymph nodes.^98^ Hypoxia also upregulates vascular endothelial growth factor (VEGF) and the interleukin‐10, which inhibit the maturation and differentiation of DCs.^99^, ^100^, ^101^ Furthermore, the secretion of large amounts of osteopontin by the hypoxic DCs promotes tumour cell migration.^102^

#### Natural killer cells

Natural killer cells are components of innate immune system and are also affected by the hypoxic TME. Hypoxia can impair the function of NK cells by downregulating the expression of several NK‐cell receptors such as NK p44, NK p30 and NK p46.^103^ Moreover, hypoxia‐induced recruitment of Tregs in the TME activates TGF‐β, an immunosuppressive cytokine that also inhibits the function of NK cells.^104^

#### Regulatory T cells

Tregs, a subset of CD4+ T cells, are responsible for immune suppression, thereby maintaining self‐tolerance and preventing autoimmunity. Hypoxia/HIF‐1α pathway is shown to upregulate the forkhead box P3 (FOXP3), a transcription factor and specific marker for Tregs, indicating that hypoxia enhances the formation of Tregs from CD4+ T cells.^91^ Hypoxia also promotes the recruitment of Tregs by inducing the chemoattractant CCL28. A positive correlation between CCL28 and HIF‐1α expression was observed in ovarian cancer, and this was linked to poor patient prognosis.^90^ Similarly, hypoxia‐induced recruitment of Tregs is found to be a negative prognostic factor in HCC and basal‐like breast cancer.^105^, ^106^

#### Myeloid‐derived suppressor cells

Myeloid‐derived suppressor cells are immature myeloid cells that undergo differentiation to mature myeloid cells in the presence of specialized cytokines. MDSCs consist of DCs, immature macrophages and granulocytes. These regulate self‐tolerance, impair T‐cell functions and are also responsible for tumour metastasis and angiogenesis.^107^ Hypoxia‐induced HIF drives the accumulation of MDSCs and blocking HIF was found to decrease the recruitment of MDSCs, tumour growth and angiogenesis.^108^

#### Tumour‐associated macrophages

Tumour‐associated macrophages (TAMs), derived from myeloid progenitors, can be classified into M1‐type (anti‐tumour and proinflammatory) and M2‐type (pro‐tumour and anti‐inflammatory) macrophages.^109^ Hypoxia via HIF‐1α mediates the differentiation of MDSCs into TAMs that prevent immune destruction by decreasing T‐cell infiltration.^110^ High number of M2‐type TAMs in the tumour region contributes to immunosuppression, tumour progression and angiogenesis, and is mostly associated with poor clinical prognosis in cancer patients.^111^ Tumour hypoxia increases the recruitment of M2‐type macrophages in the TME by inducing the expression of migratory stimulatory factors such as VEGF and endothelins.^21^ Similarly, enhanced production of lactate and the secretion of VEGF, TGF‐β, interleukin‐4 and interleukin‐6 under hypoxic conditions, create a suitable environment for the differentiation of macrophages into suppressive M2 TAMs.^112^ Moreover, hypoxia upregulates the secretion of matrix metalloproteinase (MMP7) by TAMs, which cleaves the Fas ligand from neighbouring cells, thus decreasing tumour cell lysis by T cells and NK cells.^113^, ^114^

#### Other regulatory pathways

Hypoxia attenuates anti‐tumour immunity by upregulating the expression of PD‐L1 on tumour cells, MDSCs, TAMs and DCs via HIF‐1α regulated pathways.^104^ The positive correlation and links between PD‐L1 and HIF‐1α has been reported in various studies.^115^, ^116^ HIF‐2α is also reported to increase PD‐L1 expression in clear cell renal cell carcinoma (ccRCC).^117^ Inhibition of PD‐L1 was found to promote MDSCs‐mediated T‐cell activation, thus decreasing the immunosuppressive function of MDSCs.^118^ Another checkpoint molecule overexpressed in hypoxic conditions is V‐Domain Ig Suppressor of T cell Activation (VISTA). VISTA is expressed on MDSCs, TAMS and DCs and hypoxia‐induced VISTA is shown to suppress T‐cell proliferation and function.^119^ Moreover, hypoxia upregulates the expression of cluster of differentiation 47 (CD47), a macrophage immune checkpoint protein, thus, enabling tumour cells to escape phagocytic cell death.^120^ Overexpression of CD47 has been linked to poor clinical prognosis.^121^ Furthermore, hypoxia‐induced HIF‐1α can increase the expression of metalloproteinase ADAM10, which regulates the shedding of the MHC‐I‐chain related molecule A (MICA) from the tumour cell surface. MICA is a ligand for activating the natural killer group 2, member D (NKG2D) receptor on the NK cells, and the shedding of MICA downregulates the NKG2D receptor, leading to tumour cell escape from T cells and NK cells.^122^, ^123^ Additionally, evidence suggests that hypoxic tumour cells can induce autophagy to degrade the NK‐cell derived proteases granzymes B, thus decreasing tumour cell susceptibility to NK‐cell‐mediated killing.^124^, ^125^

**Fig. 3:**
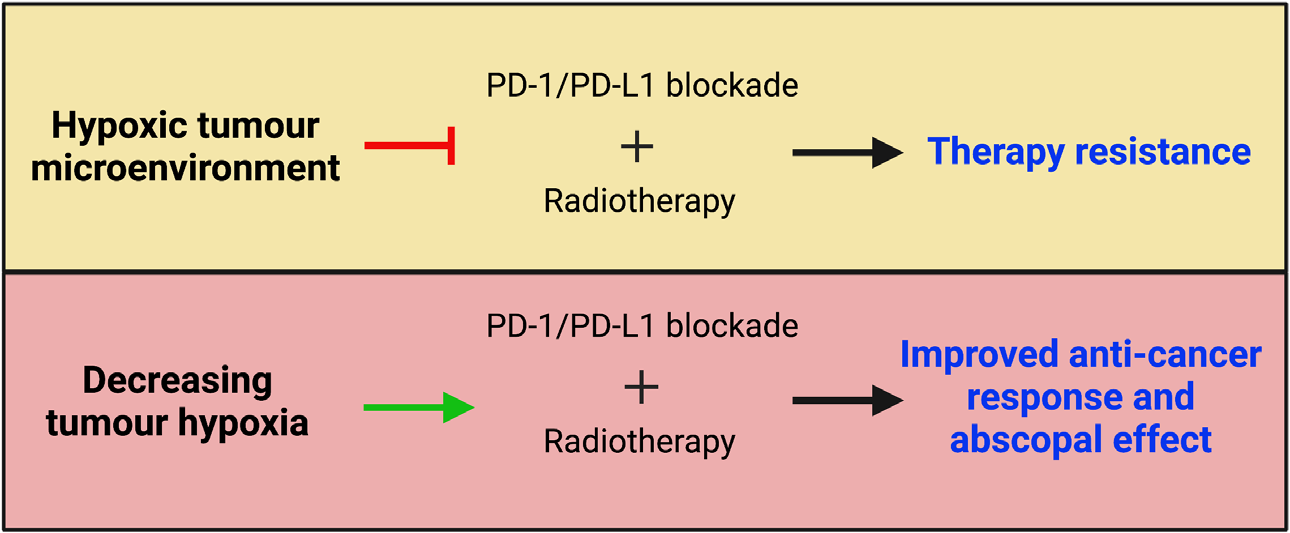
Hypoxic tumour microenvironment can act as a barrier to the effectiveness of PD‐1/PD‐L1 blockade therapy and radiotherapy. As hypoxia acts as a barrier to both radiotherapy and immunotherapy, the hypoxic tumour microenvironment (TME) may negatively influence the synergistic combination of PD‐1/PD‐L1 blockade and radiotherapy and contribute to therapy resistance. Decreasing the hypoxic TME can overcome the immunosuppressive effects mediated by hypoxia and may benefit combined PD‐1/PD‐L1 blockade therapy and radiotherapy. Incorporation of compounds that directly or indirectly target hypoxia may boost the synergistic combination of these therapies and lead to improved anti‐cancer responses and abscopal effects. Figure created using Biorender.com. [Colour figure can be viewed at wileyonlinelibrary.com]

Overall, all the above‐mentioned mechanisms indicate that the hypoxic milieu dampens the anti‐tumour immune responses and can suppress immunotherapy approaches. As hypoxia promotes radioresistance and immunosuppression,^80^, ^83^ a sustained reduction in tumour hypoxia appears to be an attractive strategy to lower immunosuppression and improve the efficacy of combined PD‐1/PD‐L1 blockade therapy and radiotherapy. Thus, the prospect of targeting hypoxia in combination with PD‐1/PD‐L1 blockade and radiotherapy appears to be encouraging (Fig. 3).

## Targeting tumour hypoxia to overcome resistance to radiotherapy and PD‐1/PD‐L1 blockade immunotherapy

### Increasing oxygen supply to the tumour

Since hypoxia develops due to lack of oxygen supply, the most relevant approach to resolve hypoxia and improve radiotherapy and immunotherapy outcomes is to increase the oxygen supply to the tumour region. Initial attempts involved the use of blood transfusions to increase haemoglobin levels and therefore improve responses to radiotherapy. However, clinical studies reported that these transfusions did not improve treatment outcomes significantly.^126^, ^127^ Other approaches that attempted to increase oxygen supply included hyperbaric oxygen breathing or the use of carbogen (95% oxygen and 5% carbon dioxide). Despite promising preclinical and early‐phase data, both approaches failed to show significant radiosensitization outcomes in patients.^82^, ^128^, ^129^ Interestingly, the combination of carbogen with nicotinamide targets hypoxia and is reported to improve radiation responses in clinical trials.^130^, ^131^ However, the temporary increase in oxygenation from radiotherapy combined with carbogen and nicotinamide maybe too short to initiate effective and durable immune responses.^132^ This suggests that other approaches are needed to result in sustained oxygenation and improve the responses to combined PD‐1/PD‐L1 blockade immunotherapy and radiotherapy.

### Hypoxia‐activated prodrugs

Hypoxia‐activated prodrugs (HAPs) have also been explored to selectivity target hypoxic tumour cells. HAPs are inactive compounds that convert to active drugs in hypoxic regions.^133^ Despite preclinical data showing cytotoxicity from various HAPs, the clinical studies of HAPs as single agent or combined with chemoradiotherapy were generally disappointing.^133^ Recently, a second‐generation HAP evofosfamide (TH‐302) has demonstrated favourable outcomes in combination with CTLA‐4 and PD‐1 blockade by curing more than 80% of tumours in a mouse prostate‐derived model. This was most likely achieved by a significant decrease in tumour hypoxia, promoting the influx of T cells and decreasing in MDSCs in the TME.^134^ Currently, a clinical trial (NCT03098160) is investigating the efficacy of TH‐302 in combination with ipilimumab (anti‐CTLA‐4) against a range of tumour types. Moreover, TH‐302 has also been assessed in combination with radiation, showing increased tumour growth delay in various preclinical tumour models.^135^, ^136^, ^137^ Research on TH‐302 is still ongoing and because previous clinical studies suggest that TH‐302 may only provide benefit to patients with high tumour hypoxic fractions, determining the hypoxia status of the patient's tumour will be beneficial before the clinical application of TH‐302.^138^ Future preclinical studies could use tumour models with high hypoxic burden to assess whether TH‐302 could improve tumour control and enhance the efficacy of combined radiotherapy and anti‐PD‐1/PD‐L1 antibodies.

### Decreasing oxygen consumption of the tumour

In contrast to the aforementioned strategies, another alternative to overcome hypoxia is to reduce tumour cells demand for oxygen by inhibiting the mitochondrial oxidative phosphorylation (OXPHOS).^139^ Increasing evidence suggests that along with active glycolysis, cancer cells also have increased mitochondrial OXPHOS, which results in increased oxygen consumption and ATP production in the tumour.^140^, ^141^, ^142^, ^143^, ^144^ OXPHOS is a metabolic pathway comprising of an electron transport chain with complexes I, II, III and IV where a series of redox reactions occur, eventually resulting in the generation of ATP at complex V. Oxygen is critical for OXPHOS as it is the terminal electron acceptor in this process.^140^ The hypothesis is that OXPHOS inhibition will decrease the oxygen consumption rate (OCR) of tumour cells, allowing for the diffusion of unmetabolized oxygen into hypoxic regions, reserving more available oxygen, and thus reducing tumour hypoxia. Mathematical modelling also indicates that decreasing cellular oxygen consumption appears to be a more efficient method to alleviate tumour hypoxia than increasing oxygen supply.^139^

The assumptions underlying the use of OXPHOS inhibitors are that elevated oxidative metabolism in cancers is associated with increased tumour hypoxia, which impedes the success of radiotherapy and immunotherapy. Indeed, several studies have now demonstrated that OXPHOS inhibitors could decrease OCR and tumour hypoxia. Metformin, an antidiabetic drug and a mitochondrial complex I inhibitor, is the first compound tested in this context. It decreased tumour hypoxia and improved tumour radiation response in colorectal and brain tumour preclinical models.^145^, ^146^ As a result, metformin has been evaluated in clinical trials as a potential radiosensitizer.^147^ In another study, metformin‐induced reduction in tumour hypoxia enhanced the effect of PD‐1 blockade immunotherapy by improving the function of cytotoxic T cells.^148^ Another compound, atovaquone, an anti‐malarial drug and a mitochondrial complex III inhibitor, was found to reduce the OCR in numerous cancer cell lines.^149^ It alleviated tumour hypoxia in colorectal and hypopharyngeal carcinoma xenograft models and caused a significant tumour growth delay in combination with radiation.^149^ In a recent study, atovaquone/albumin nanoparticles combined with anti‐PD‐1 antibody significantly improved tumour control in mice xenograft models. The observed effect was due to atovaquone‐mediated hypoxia reduction, leading to increased recruitment of CD8+ T cells, thereby enhancing the efficacy of anti‐PD‐1 immunotherapy.^150^ The findings using metformin and atovaquone combined with an anti‐PD‐1 antibody provide convincing evidence that modification of the hypoxic TME using OXPHOS inhibitors could improve the efficacy of immunotherapy. Additionally, the hypoxia modification efficacy of atovaquone was corroborated in a recent clinical trial (NCT02628080) showing increased tumour oxygenation and inhibition of hypoxic gene expression in NSCLC patients.^151^ These promising findings have initiated another ongoing trial testing the efficacy of atovaquone in combination with chemoradiotherapy (NCT04648033). Furthermore, papaverine, an antispasmodic drug and a mitochondrial complex I inhibitor, has provided further support towards hypoxia inhibition by OXPHOS inhibitors. This drug also reduced OCR *in vitro*, decreased tumour hypoxia and enhanced radiation response in *in vivo* breast and lung cancer models.^152^ Collectively, these findings provide a strong rationale to investigate OXPHOS inhibitors to eliminate the hypoxic TME and improve the efficacies of PD‐1/PD‐L1 blockade and radiotherapy.

Various studies have supported the effectiveness of OXPHOS inhibition in targeting cancers with upregulated OXPHOS. It also appears that cancers with increased oxidative metabolism might be resistant to PD‐1/PD‐L1 blockade immunotherapy. As an example, a study on melanoma patients reported that increased oxidative metabolism could act as a barrier to successful PD‐1 blockade therapy.^153^ Interestingly, transcriptional profiling data revealed enrichment of hypoxia gene sets in non‐responders to anti‐PD‐1 therapy compared with responders, further supporting the notion that tumour hypoxia promotes resistance to anti‐PD‐1 immunotherapy.^154^, ^155^ Similarly, links between elevated OXPHOS and immune checkpoint therapy resistance are reported by another study showing that PD‐1 resistant NSCLC model appeared to have higher oxidative metabolism than PD‐1 sensitive model.^156^ This preclinical study highlighted the significance of combining IACS‐010759 (mitochondrial complex I inhibitor) with radiotherapy to overcome PD‐1 resistance. The addition of IACS‐010759 to the combination of radiotherapy and anti‐PD‐1 antibody significantly prolonged survival and increased abscopal responses in the PD‐1 resistant model. Furthermore, they demonstrated that IACS‐010759 increases anti‐tumour immunity by decreasing Tregs and increasing granzyme B+ CD8+ T cells.^156^ These results suggest that IACS‐010759 alleviates tumour hypoxia by blocking OXPHOS and improves anti‐tumour response by overcoming radiation resistance and the immunosuppressive TME. The triple combination of OXPHOS inhibition, radiotherapy and anti‐PD‐1 checkpoint immunotherapy is assessed for the first time by this study. It provides valuable insight into targeting the hypoxic TME to improve the synergistic combination of PD‐1/PD‐L1 blockade and radiotherapy and increase the abscopal effects (Fig. 3). Future investigations on combined PD‐1/PD‐L1 blockade and radiotherapy could consider assessing other OXPHOS inhibitors or compounds that are found to decrease the OCR, influence tumour hypoxia and modulate radiosensitivity. Some of these include the mitochondrial complex inhibitors (metformin, atovaquone and papaverine), non‐steroidal anti‐inflammatory drugs, glucocorticoids, antiangiogenic agents and MAPK inhibitors (reviewed in Ref. 157).

### Inhibiting HIF activity

Hypoxia‐inducible factor inhibitors combined with radiotherapy and PD‐1/PD‐L1 blockade may also prove to be an effective anti‐cancer strategy. HIF increases the expression of PD‐L1 and has numerous effects on the immune system, summarized above. HIF also has important roles in the tumour in driving metastasis, angiogenesis and tumour metabolism. Of the two main targets, HIF‐1 and HIF‐2, only a direct HIF‐2 inhibitor, belzutifan, has been approved for clinical use.^158^ Belzutifan is currently undergoing clinical assessment in combination with the anti‐PD‐1 antibody pembrolizumab and other compounds on ccRCC (NCT04736706). There are several HIF‐1 inhibitors and clinical trials that have been reviewed elsewhere.^159^, ^160^ While space constraints limit our discussion on the topic, preclinical studies indicate that combining HIF inhibition with immune checkpoint inhibitors can promote anti‐tumour immunity in various cancers.^161^, ^162^ Moreover, drugs including PX‐478, bortezomib and chetomin that could indirectly inhibit HIF‐1 protein expression have demonstrated radiosensitization in several cancers at preclinical level.^163^, ^164^, ^165^, ^166^, ^167^ Combinations of HIF inhibitors, radiotherapy and PD‐1/PD‐L1 blockade is a growing area of interest, and further studies will determine whether this is a viable strategy.

## Conclusion

The combination of PD‐1/PD‐L1 blockade and radiotherapy is a promising development in cancer management; however, the complexity of inherent and acquired resistance to both these therapies can limit their effectiveness in the clinical settings. Evidence from literature suggests that hypoxic TME could impede the success of both radiotherapy and immunotherapy, and several studies have proved that inhibition of hypoxia can sensitize tumour cells to radiotherapy and improve immunotherapy outcomes. This suggests that hypoxia could also be exploited to improve the synergistic combination of PD‐1/PD‐L1 blockade and radiotherapy. Compounds that have shown promising reduction in tumour hypoxia could be investigated along with the combination of PD‐1/PD‐L1 blockade and radiotherapy. There is still need for more preclinical and clinical studies to address which of the hypoxia modifying compounds could boost the anti‐tumour immunity without suppressing it to achieve optimal therapeutic results when combined with PD‐1/PD‐L1 blockade and radiotherapy.

## References

[1] Sanmamed MF , Chen L . A paradigm shift in cancer immunotherapy: from enhancement to normalization. Cell 2018; 175: 313–26. 30290139 10.1016/j.cell.2018.09.035 PMC6538253

[2] Sun J‐Y , Lu X‐J . Cancer immunotherapy: current applications and challenges. Cancer Lett 2020; 480: 1–3. 32229188 10.1016/j.canlet.2020.03.024

[3] Pardoll D . Cancer and the immune system: basic concepts and targets for intervention. Semin Oncol 2015; 42: 523–38. 26320058 10.1053/j.seminoncol.2015.05.003 PMC5595144

[4] Jiang Y , Chen M , Nie H , Yuan Y . PD‐1 and PD‐L1 in cancer immunotherapy: clinical implications and future considerations. Hum Vaccines Immunother 2019; 15: 1111–22. 10.1080/21645515.2019.1571892 PMC6605868 30888929

[5] Buchbinder EI , Desai A . CTLA‐4 and PD‐1 pathways similarities, differences, and implications of their inhibition. Am J Clin Oncol 2016; 39: 98–106. 26558876 10.1097/COC.0000000000000239 PMC4892769

[6] Egen JG , Ouyang W , Wu LC . Human anti‐tumor immunity: insights from immunotherapy clinical trials. Immunity 2020; 52: 36–54. 31940272 10.1016/j.immuni.2019.12.010

[7] Motzer RJ , Escudier B , McDermott DF *et al*. Nivolumab versus everolimus in advanced renal‐cell carcinoma. N Engl J Med 2015; 373: 1803–13. 26406148 10.1056/NEJMoa1510665 PMC5719487

[8] Robert C , Schachter J , Long GV *et al*. Pembrolizumab versus ipilimumab in advanced melanoma. N Engl J Med 2015; 372: 2521–32. 25891173 10.1056/NEJMoa1503093

[9] Chow LQM , Haddad R , Gupta S *et al*. Antitumor activity of pembrolizumab in biomarker‐unselected patients with recurrent and/or metastatic head and neck squamous cell carcinoma: results from the phase Ib KEYNOTE‐012 expansion cohort. J Clin Oncol 2016; 34: 3838–45. 27646946 10.1200/JCO.2016.68.1478 PMC6804896

[10] Bellmunt J , de Wit R , Vaughn DJ *et al*. Pembrolizumab as second‐line therapy for advanced urothelial carcinoma. N Engl J Med 2017; 376: 1015–26. 28212060 10.1056/NEJMoa1613683 PMC5635424

[11] Overman MJ , McDermott R , Leach JL *et al*. Nivolumab in patients with metastatic DNA mismatch repair‐deficient or microsatellite instability‐high colorectal cancer (CheckMate 142): an open‐label, multicentre, phase 2 study. Lancet Oncol 2017; 18: 1182–91. 28734759 10.1016/S1470-2045(17)30422-9 PMC6207072

[12] Sharma PP , Retz MP , Siefker‐Radtke AMD *et al*. Nivolumab in metastatic urothelial carcinoma after platinum therapy (CheckMate 275): a multicentre, single‐arm, phase 2 trial. Lancet Oncol 2017; 18: 312–22. 28131785 10.1016/S1470-2045(17)30065-7

[13] El‐Khoueiry ABD , Sangro BMD , Yau TMD *et al*. Nivolumab in patients with advanced hepatocellular carcinoma (CheckMate 040): an open‐label, non‐comparative, phase 1/2 dose escalation and expansion trial. Lancet 2017; 389: 2492–502. 28434648 10.1016/S0140-6736(17)31046-2 PMC7539326

[14] Rosner S , Reuss JE , Forde PM . PD‐1 blockade in early‐stage lung cancer. Annu Rev Med 2019; 70: 425–35. 30355264 10.1146/annurev-med-050217-025205

[15] Hamid O , Robert C , Daud A *et al*. Five‐year survival outcomes for patients with advanced melanoma treated with pembrolizumab in KEYNOTE‐001. Ann Oncol 2019; 30: 582–8. 30715153 10.1093/annonc/mdz011 PMC6503622

[16] Bai J , Gao Z , Li X , Dong L , Han W , Nie J . Regulation of PD‐1/PD‐L1 pathway and resistance to PD‐1/PD‐L1 blockade. Oncotarget 2017; 8: 110693–707. 29299180 10.18632/oncotarget.22690 PMC5746415

[17] Lei Q , Wang D , Sun K , Wang L , Zhang Y . Resistance mechanisms of anti‐PD1/PDL1 therapy in solid tumors. Front Cell Dev Biol 2020; 8: 672. 32793604 10.3389/fcell.2020.00672 PMC7385189

[18] Ott PA , Bang Y‐J , Piha‐Paul SA *et al*. T‐cell–inflamed gene‐expression profile, programmed death ligand 1 expression, and tumor mutational burden predict efficacy in patients treated with pembrolizumab across 20 cancers: KEYNOTE‐028. J Clin Oncol 2019; 37: 318–27. 30557521 10.1200/JCO.2018.78.2276

[19] Cristescu R , Mogg R , Ayers M *et al*. Pan‐tumor genomic biomarkers for PD‐1 checkpoint blockade‐based immunotherapy. Science 2018; 362: 197. 10.1126/science.aar3593 PMC6718162 30309915

[20] Sharma P , Allison JP . The future of immune checkpoint therapy. Science 2015; 348: 56–61. 25838373 10.1126/science.aaa8172

[21] Fu Z , Mowday AM , Smaill JB , Hermans IF , Patterson AV . Tumour hypoxia‐mediated immunosuppression: mechanisms and therapeutic approaches to improve cancer immunotherapy. Cell 2021; 10: 1006. 10.3390/cells10051006 PMC8146304 33923305

[22] Wang B , Zhao Q , Zhang Y *et al*. Targeting hypoxia in the tumor microenvironment: a potential strategy to improve cancer immunotherapy. J Exp Clin Cancer Res 2021; 40: 24. 33422072 10.1186/s13046-020-01820-7 PMC7796640

[23] Patel SP , Kurzrock R . PD‐L1 expression as a predictive biomarker in cancer immunotherapy. Mol Cancer Ther 2015; 14: 847–56. 25695955 10.1158/1535-7163.MCT-14-0983

[24] Hino R , Kabashima K , Kato Y *et al*. Tumor cell expression of programmed cell death‐1 ligand 1 is a prognostic factor for malignant melanoma. Cancer 2010; 116: 1757–66. 20143437 10.1002/cncr.24899

[25] Zou W , Chen L . Inhibitory B7‐family molecules in the tumour microenvironment. Nat Rev Immunol 2008; 8: 467–77. 18500231 10.1038/nri2326

[26] Taube JM , Klein A , Brahmer JR *et al*. Association of PD‐1, PD‐1 ligands, and other features of the tumor immune microenvironment with response to anti‐PD‐1 therapy. Clin Cancer Res 2014; 20: 5064–74. 24714771 10.1158/1078-0432.CCR-13-3271 PMC4185001

[27] Yearley JH , Gibson C , Yu N *et al*. PD‐L2 expression in human tumors: relevance to anti‐PD‐1 therapy in cancer. Clin Cancer Res 2017; 23: 3158–67. 28619999 10.1158/1078-0432.CCR-16-1761

[28] Shi F , Shi M , Zeng Z *et al*. PD‐1 and PD‐L1 upregulation promotes CD8+ T‐cell apoptosis and postoperative recurrence in hepatocellular carcinoma patients. Int J Cancer 2011; 128: 887–96. 20473887 10.1002/ijc.25397

[29] Patsoukis N , Sari D , Boussiotis VA . PD‐1 inhibits T cell proliferation by upregulating p27 and p15 and suppressing Cdc25A. Cell Cycle 2012; 11: 4305–9. 23032366 10.4161/cc.22135 PMC3552912

[30] Wherry EJ . T cell exhaustion. Nat Immunol 2011; 12: 492–9. 21739672 10.1038/ni.2035

[31] Pauken KE , Wherry EJ . Overcoming T cell exhaustion in infection and cancer. Trends Immunol 2015; 36: 265–76. 25797516 10.1016/j.it.2015.02.008 PMC4393798

[32] Borrego‐Soto G , Ortiz‐López R , Rojas‐Martínez A . Ionizing radiation‐induced DNA injury and damage detection in patients with breast cancer. Genet Mol Biol 2015; 38: 420–32. 26692152 10.1590/S1415-475738420150019 PMC4763322

[33] Schaue DPD , Ratikan JAMS , Iwamoto KSPD , McBride WHDS . Maximizing tumor immunity with fractionated radiation. Int J Radiat Oncol Biol Phys 2012; 83: 1306–10. 22208977 10.1016/j.ijrobp.2011.09.049 PMC3337972

[34] Schaue D , Comin‐Anduix B , Ribas A *et al*. T‐cell responses to survivin in cancer patients undergoing radiation therapy. Clin Cancer Res 2008; 14: 4883–90. 18676762 10.1158/1078-0432.CCR-07-4462 PMC2748652

[35] Deng L , Liang H , Xu M *et al*. STING‐dependent cytosolic DNA sensing promotes radiation‐induced type I interferon‐dependent antitumor immunity in immunogenic tumors. Immunity 2014; 41: 843–52. 25517616 10.1016/j.immuni.2014.10.019 PMC5155593

[36] Obeid M , Tesniere A , Ghiringhelli F *et al*. Calreticulin exposure dictates the immunogenicity of cancer cell death. Nat Med 2007; 13: 54–61. 17187072 10.1038/nm1523

[37] Apetoh L , Ghiringhelli F , Tesniere A *et al*. Toll‐like receptor 4‐dependent contribution of the immune system to anticancer chemotherapy and radiotherapy. Nat Med 2007; 13: 1050–9. 17704786 10.1038/nm1622

[38] Ghiringhelli F , Apetoh L , Tesniere A *et al*. Activation of the NLRP3 inflammasome in dendritic cells induces IL‐1beta‐dependent adaptive immunity against tumors. Nat Med 2009; 15: 1170–8. 19767732 10.1038/nm.2028

[39] Garnett CT , Palena C , Chakarborty M , Tsang K‐Y , Schlom J , Hodge JW . Sublethal irradiation of human tumor cells modulates phenotype resulting in enhanced killing by cytotoxic T lymphocytes. Cancer Res 2004; 64: 7985–94. 15520206 10.1158/0008-5472.CAN-04-1525

[40] Waring P , Müllbacher A . Cell death induced by the Fas/Fas ligand pathway and its role in pathology. Immunol Cell Biol 1999; 77: 312–7. 10457197 10.1046/j.1440-1711.1999.00837.x

[41] Reits EA , Hodge JW , Herberts CA *et al*. Radiation modulates the peptide repertoire, enhances MHC class I expression, and induces successful antitumor immunotherapy. J Exp Med 2006; 203: 1259–71. 16636135 10.1084/jem.20052494 PMC3212727

[42] Demaria S , Golden EB , Formenti SC . Role of local radiation therapy in cancer immunotherapy. JAMA Oncol 2015; 1: 1325–32. 26270858 10.1001/jamaoncol.2015.2756

[43] Kang C , Jeong S‐Y , Song SY , Choi EK . The emerging role of myeloid‐derived suppressor cells in radiotherapy. Radiat Oncol J 2020; 38: 1–10. 32229803 10.3857/roj.2019.00640 PMC7113146

[44] Liu S , Sun X , Luo J *et al*. Effects of radiation on T regulatory cells in normal states and cancer: mechanisms and clinical implications. Am J Cancer Res 2015; 5: 3276–85. 26807310 PMC4697676

[45] Tsai C‐SMD , Chen F‐HMS , Wang C‐CMDPD *et al*. Macrophages from irradiated tumors express higher levels of iNOS, arginase‐I and COX‐2, and promote tumor growth. Int J Radiat Oncol Biol Phys 2007; 68: 499–507. 17398016 10.1016/j.ijrobp.2007.01.041

[46] Barcellos‐Hoff MH , Derynck R , Tsang MLS , Weatherbee JA . Transforming growth factor‐β activation in irradiated murine mammary gland. J Clin Investig 1994; 93: 892–9. 8113421 10.1172/JCI117045 PMC293960

[47] Vanpouille‐Box C , Diamond JM , Pilones KA *et al*. TGFβ is a master regulator of radiation therapy‐induced antitumor immunity. Cancer Res 2015; 75: 2232–42. 25858148 10.1158/0008-5472.CAN-14-3511 PMC4522159

[48] Reynders K , Illidge T , Siva S , Chang JY , De Ruysscher D . The abscopal effect of local radiotherapy: using immunotherapy to make a rare event clinically relevant. Cancer Treat Rev 2015; 41: 503–10. 25872878 10.1016/j.ctrv.2015.03.011 PMC4816218

[49] Demaria S , Ng B , Devitt ML *et al*. Ionizing radiation inhibition of distant untreated tumors (abscopal effect) is immune mediated. Int J Radiat Oncol Biol Phys 2004; 58: 862–70. 14967443 10.1016/j.ijrobp.2003.09.012

[50] Nobler MP . The abscopal effect in malignant lymphoma and its relationship to lymphocyte circulation. Radiology 1969; 93: 410–2. 5822721 10.1148/93.2.410

[51] Formenti SCD , Demaria SMD . Systemic effects of local radiotherapy. Lancet Oncol 2009; 10: 718–26. 19573801 10.1016/S1470-2045(09)70082-8 PMC2782943

[52] Zeng JMD , See APBS , Phallen JBS *et al*. Anti‐PD‐1 blockade and stereotactic radiation produce long‐term survival in mice with intracranial gliomas. Int J Radiat Oncol Biol Phys 2013; 86: 343–9. 23462419 10.1016/j.ijrobp.2012.12.025 PMC3963403

[53] Sharabi AB , Nirschl CJ , Kochel CM *et al*. Stereotactic radiation therapy augments antigen‐specific PD‐1‐mediated antitumor immune responses via cross‐presentation of tumor antigen. Cancer Immunol Res 2015; 3: 345–55. 25527358 10.1158/2326-6066.CIR-14-0196 PMC4390444

[54] Deng L , Liang H , Burnette B *et al*. Irradiation and anti‐PD‐L1 treatment synergistically promote antitumor immunity in mice. J Clin Investig 2014; 124: 687–95. 24382348 10.1172/JCI67313 PMC3904601

[55] Dovedi SJ , Adlard AL , Lipowska‐Bhalla G *et al*. Acquired resistance to fractionated radiotherapy can be overcome by concurrent PD‐L1 blockade. Cancer Res 2014; 74: 5458–68. 25274032 10.1158/0008-5472.CAN-14-1258

[56] Wang X , Schoenhals JE , Li A *et al*. Suppression of type I IFN signaling in tumors mediates resistance to anti‐PD‐1 treatment that can be overcome by radiotherapy. Cancer Res 2017; 77: 839–50. 27821490 10.1158/0008-5472.CAN-15-3142 PMC5875182

[57] Maity A , Mick R , Huang AC *et al*. A phase I trial of pembrolizumab with hypofractionated radiotherapy in patients with metastatic solid tumours. Br J Cancer 2018; 119: 1200–7. 30318516 10.1038/s41416-018-0281-9 PMC6251028

[58] Theelen WSME , Peulen HMU , Lalezari F *et al*. Effect of pembrolizumab after stereotactic body radiotherapy vs pembrolizumab alone on tumor response in patients with advanced non–small cell lung cancer: results of the PEMBRO‐RT phase 2 randomized clinical trial. JAMA Oncol 2019; 5: 1276–82. 31294749 10.1001/jamaoncol.2019.1478 PMC6624814

[59] Shaverdian NMD , Lisberg AEMD , Bornazyan KMD *et al*. Previous radiotherapy and the clinical activity and toxicity of pembrolizumab in the treatment of non‐small‐cell lung cancer: a secondary analysis of the KEYNOTE‐001 phase 1 trial. Lancet Oncol 2017; 18: 895–903. 28551359 10.1016/S1470-2045(17)30380-7 PMC5538772

[60] Antonia SJ , Villegas A , Daniel D *et al*. Durvalumab after chemoradiotherapy in stage III non–small‐cell lung cancer. N Engl J Med 2017; 377: 1919–29. 28885881 10.1056/NEJMoa1709937

[61] Gong J , Le TQ , Massarelli E , Hendifar AE , Tuli R . Radiation therapy and PD‐1/PD‐L1 blockade: the clinical development of an evolving anticancer combination. J Immunother Cancer 2018; 6: 46. 29866197 10.1186/s40425-018-0361-7 PMC5987486

[62] Cao Y , Li W , Wang Z , Pang H . Potential and unsolved problems of anti‐PD‐1/PD‐L1 therapy combined with radiotherapy. Tumori J 2021; 107: 282–91. 10.1177/0300891620940382 32734832

[63] Jabbour SK , Berman AT , Decker RH *et al*. Phase 1 trial of pembrolizumab administered concurrently with chemoradiotherapy for locally advanced non–small cell lung cancer: a nonrandomized controlled trial. JAMA Oncol 2020; 6: 848–55. 32077891 10.1001/jamaoncol.2019.6731 PMC7042914

[64] Sundahl N , Vandekerkhove G , Decaestecker K *et al*. Randomized phase 1 trial of pembrolizumab with sequential versus concomitant stereotactic body radiotherapy in metastatic urothelial carcinoma. Eur Urol 2019; 75: 707–11. 30665814 10.1016/j.eururo.2019.01.009

[65] Vanpouille‐Box C , Alard A , Aryankalayil MJ *et al*. DNA exonuclease Trex1 regulates radiotherapy‐induced tumour immunogenicity. Nat Commun 2017; 8: 15618. 28598415 10.1038/ncomms15618 PMC5472757

[66] Dewan MZ , Galloway AE , Kawashima N *et al*. Fractionated but not single‐dose radiotherapy induces an immune‐mediated abscopal effect when combined with anti–CTLA‐4 antibody. Clin Cancer Res 2009; 15: 5379–88. 19706802 10.1158/1078-0432.CCR-09-0265 PMC2746048

[67] Vaupel P , Höckel M , Mayer A . Detection and characterization of tumor hypoxia using pO2 histography. Antioxid Redox Signal 2007; 9: 1221–35. 17536958 10.1089/ars.2007.1628

[68] McKeown SR . Defining normoxia, physoxia and hypoxia in tumours‐implications for treatment response. Br J Radiol 2014; 87: 20130676. 24588669 10.1259/bjr.20130676 PMC4064601

[69] Nagy JA , Chang S‐H , Shih S‐C , Dvorak AM , Dvorak HF . Heterogeneity of the tumor vasculature. Semin Thromb Hemost 2010; 36: 321–31. 20490982 10.1055/s-0030-1253454 PMC3278036

[70] Baluk P , Hashizume H , McDonald DM . Cellular abnormalities of blood vessels as targets in cancer. Curr Opin Genet Dev 2005; 15: 102–11. 15661540 10.1016/j.gde.2004.12.005

[71] Pennacchietti S , Michieli P , Galluzzo M , Mazzone M , Giordano S , Comoglio PM . Hypoxia promotes invasive growth by transcriptional activation of the met protooncogene. Cancer Cell 2003; 3: 347–61. 12726861 10.1016/s1535-6108(03)00085-0

[72] Erler JT , Cawthorne CJ , Williams KJ *et al*. Hypoxia‐mediated down‐regulation of Bid and Bax in tumors occurs via hypoxia‐inducible factor 1‐dependent and independent mechanisms and contributes to drug resistance. Mol Cell Biol 2004; 24: 2875–89. 15024076 10.1128/MCB.24.7.2875-2889.2004 PMC371100

[73] Mak TW , Cairns RA , Harris IS . Regulation of cancer cell metabolism. Nat Rev Cancer 2011; 11: 85–95. 21258394 10.1038/nrc2981

[74] Lunt SJ , Chaudary N , Hill RP . The tumor microenvironment and metastatic disease. Clin Exp Metastasis 2008; 26: 19–34. 18543068 10.1007/s10585-008-9182-2

[75] Birner P , Schindl M , Obermair A , Plank C , Breitenecker G , Oberhuber G . Overexpression of hypoxia‐inducible factor 1α is a marker for an unfavorable prognosis in early‐stage invasive cervical cancer. Cancer Res 2000; 60: 4693–6. 10987269

[76] Giatromanolaki A , Koukourakis MI , Sivridis E *et al*. Relation of hypoxia inducible factor 1α and 2α in operable non‐small cell lung cancer to angiogenic/molecular profile of tumours and survival. Br J Cancer 2001; 85: 881–90. 11556841 10.1054/bjoc.2001.2018 PMC2375073

[77] Schindl M , Schoppmann SF , Hausmaninger H *et al*. Overexpression of hypoxia‐inducible factor 1α is associated with an unfavorable prognosis in lymph node‐positive breast cancer. Clin Cancer Res 2002; 8: 1831–7. 12060624

[78] Aebersold DM , Burri P , Beer KT *et al*. Expression of hypoxia‐inducible factor‐1α: a novel predictive and prognostic parameter in the radiotherapy of oropharyngeal cancer. Cancer Res 2001; 61: 2911–6. 11306467

[79] Huang R , Zhou P‐K . HIF‐1 signaling: a key orchestrator of cancer radioresistance. Radiat Med Prot 2020; 1: 7–14.

[80] Noman MZ , Hasmim M , Lequeux A *et al*. Improving cancer immunotherapy by targeting the hypoxic tumor microenvironment: new opportunities and challenges. Cell 2019; 8: 1083. 10.3390/cells8091083 PMC6770817 31540045

[81] Rey S , Schito L , Koritzinsky M , Wouters BG . Molecular targeting of hypoxia in radiotherapy. Adv Drug Deliv Rev 2017; 109: 45–62. 27771366 10.1016/j.addr.2016.10.002

[82] Tharmalingham H , Hoskin P . Clinical trials targeting hypoxia. Br J Radiol 2019; 92: 20170966. 29979089 10.1259/bjr.20170966 PMC6435072

[83] Wilson WR , Hay MP . Targeting hypoxia in cancer therapy. Nat Rev Cancer 2011; 11: 393–410. 21606941 10.1038/nrc3064

[84] Lequeux A , Noman MZ , Xiao M *et al*. Impact of hypoxic tumor microenvironment and tumor cell plasticity on the expression of immune checkpoints. Cancer Lett 2019; 458: 13–20. 31136782 10.1016/j.canlet.2019.05.021

[85] Daniel SK , Sullivan KM , Labadie KP , Pillarisetty VG . Hypoxia as a barrier to immunotherapy in pancreatic adenocarcinoma. Clin Transl Med 2019; 8: 1–17. 30931508 10.1186/s40169-019-0226-9 PMC6441665

[86] Chouaib S , Messai Y , Couve S , Escudier B , Hasmim M , Noman MZ . Hypoxia promotes tumor growth in linking angiogenesis to immune escape. Front Immunol 2012; 3: 21. 22566905 10.3389/fimmu.2012.00021 PMC3341970

[87] Abou Khouzam R , Goutham HV , Zaarour RF *et al*. Integrating tumor hypoxic stress in novel and more adaptable strategies for cancer immunotherapy. Semin Cancer Biol 2020; 65: 140–54. 31927131 10.1016/j.semcancer.2020.01.003

[88] Mpekris F , Voutouri C , Baish JW *et al*. Combining microenvironment normalization strategies to improve cancer immunotherapy. Proc Natl Acad Sci USA 2020; 117: 3728–37. 32015113 10.1073/pnas.1919764117 PMC7035612

[89] Ohta A , Diwanji R , Kini R , Subramanian M , Ohta A , Sitkovsky M . In vivo T cell activation in lymphoid tissues is inhibited in the oxygen‐poor microenvironment. Front Immunol 2011; 2: 27. 22566817 10.3389/fimmu.2011.00027 PMC3342240

[90] Facciabene A , Peng X , Hagemann IS *et al*. Tumour hypoxia promotes tolerance and angiogenesis via CCL28 and T reg cells. Nature 2011; 475: 226–30. 21753853 10.1038/nature10169

[91] Clambey ET , McNamee EN , Westrich JA *et al*. Hypoxia‐inducible factor‐1 alpha–dependent induction of FoxP3 drives regulatory T‐cell abundance and function during inflammatory hypoxia of the mucosa. Proc Natl Acad Sci USA 2012; 109: E2784–93. 22988108 10.1073/pnas.1202366109 PMC3478644

[92] Nakagawa Y , Negishi Y , Shimizu M , Takahashi M , Ichikawa M , Takahashi H . Effects of extracellular pH and hypoxia on the function and development of antigen‐specific cytotoxic T lymphocytes. Immunol Lett 2015; 167: 72–86. 26209187 10.1016/j.imlet.2015.07.003

[93] Fischer K , Hoffmann P , Voelkl S *et al*. Inhibitory effect of tumor cell–derived lactic acid on human T cells. Blood 2007; 109: 3812–9. 17255361 10.1182/blood-2006-07-035972

[94] Casanello P , Torres A , Sanhueza F *et al*. Equilibrative nucleoside transporter 1 expression is downregulated by hypoxia in human umbilical vein endothelium. Circ Res 2005; 97: 16–24. 15933265 10.1161/01.RES.0000172568.49367.f8

[95] Hammami A , Allard D , Allard B , Stagg J . Targeting the adenosine pathway for cancer immunotherapy. Semin Immunol 2019; 42: 101304. 31604539 10.1016/j.smim.2019.101304

[96] Hatfield SM , Kjaergaard J , Lukashev D *et al*. Immunological mechanisms of the antitumor effects of supplemental oxygenation. Sci Transl Med 2015; 7: 277ra30. 10.1126/scitranslmed.aaa1260 PMC4641038 25739764

[97] Correale P , Rotundo MS , Botta C *et al*. Tumor infiltration by T lymphocytes expressing chemokine receptor 7 (CCR7) is predictive of favorable outcome in patients with advanced colorectal carcinoma. Clin Cancer Res 2012; 18: 850–7. 22142823 10.1158/1078-0432.CCR-10-3186

[98] Mancino A , Schioppa T , Larghi P *et al*. Divergent effects of hypoxia on dendritic cell functions. Blood 2008; 112: 3723–34. 18694997 10.1182/blood-2008-02-142091

[99] Allavena P , Piemonti L , Longoni D *et al*. IL‐10 prevents the differentiation of monocytes to dendritic cells but promotes their maturation to macrophages. Eur J Immunol 1998; 28: 359–69. 9485215 10.1002/(SICI)1521-4141(199801)28:01<359::AID-IMMU359>3.0.CO;2-4

[100] Oyama T , Ran S , Ishida T *et al*. Vascular endothelial growth factor affects dendritic cell maturation through the inhibition of nuclear factor‐κB activation in hemopoietic progenitor cells. J Immunol 1998; 160: 1224–32. 9570538

[101] Takayama T , Morelli AE , Onai N *et al*. Mammalian and viral IL‐10 enhance C‐C chemokine receptor 5 but down‐regulate C‐C chemokine receptor 7 expression by myeloid dendritic cells: impact on chemotactic responses and in vivo homing ability. J Immunol 2001; 166: 7136–43. 11390459 10.4049/jimmunol.166.12.7136

[102] Yang M , Ma C , Liu S *et al*. HIF‐dependent induction of adenosine receptor A2b skews human dendritic cells to a Th2‐stimulating phenotype under hypoxia. Immunol Cell Biol 2010; 88: 165–71. 19841638 10.1038/icb.2009.77

[103] Balsamo M , Manzini C , Pietra G *et al*. Hypoxia downregulates the expression of activating receptors involved in NK‐cell‐mediated target cell killing without affecting ADCC. Eur J Immunol 2013; 43: 2756–64. 23913266 10.1002/eji.201343448

[104] Labiano S , Palazon A , Melero I . Immune response regulation in the tumor microenvironment by hypoxia. Semin Oncol 2015; 42: 378–86. 25965356 10.1053/j.seminoncol.2015.02.009

[105] Ren L , Yu Y , Wang L , Zhu Z , Lu R , Yao Z . Hypoxia‐induced CCL28 promotes recruitment of regulatory T cells and tumor growth in liver cancer. Oncotarget 2016; 7: 75763–73. 27716621 10.18632/oncotarget.12409 PMC5342776

[106] Yan M , Jene N , Byrne D *et al*. Recruitment of regulatory T cells is correlated with hypoxia‐induced CXCR4 expression, and is associated with poor prognosis in basal‐like breast cancers. Breast Cancer Res 2011; 13: R47. 21521526 10.1186/bcr2869 PMC3219210

[107] Hou A , Hou K , Huang Q , Lei Y , Chen W . Targeting myeloid‐derived suppressor cell, a promising strategy to overcome resistance to immune checkpoint inhibitors. Front Immunol 2020; 11: 783. 32508809 10.3389/fimmu.2020.00783 PMC7249937

[108] Chiu DKC , Xu IMJ , Lai RKH *et al*. Hypoxia induces myeloid‐derived suppressor cell recruitment to hepatocellular carcinoma through chemokine (C‐C motif) ligand 26. Hepatology 2016; 64: 797–813. 27228567 10.1002/hep.28655

[109] Vitale I , Manic G , Coussens LM , Kroemer G , Galluzzi L . Macrophages and metabolism in the tumor microenvironment. Cell Metab 2019; 30: 36–50. 31269428 10.1016/j.cmet.2019.06.001

[110] Corzo CA , Condamine T , Lu L *et al*. HIF‐1α regulates function and differentiation of myeloid‐derived suppressor cells in the tumor microenvironment. J Exp Med 2010; 207: 2439–53. 20876310 10.1084/jem.20100587 PMC2964584

[111] Komohara Y , Fujiwara Y , Ohnishi K , Takeya M . Tumor‐associated macrophages: potential therapeutic targets for anti‐cancer therapy. Adv Drug Deliv Rev 2016; 99 (Pt B): 180–5. 26621196 10.1016/j.addr.2015.11.009

[112] Chouaib S , Noman MZ , Kosmatopoulos K , Curran MA . Hypoxic stress: obstacles and opportunities for innovative immunotherapy of cancer. Oncogene 2017; 36: 439–45. 27345407 10.1038/onc.2016.225 PMC5937267

[113] Noman MZ , Hasmim M , Messai Y *et al*. Hypoxia: a key player in antitumor immune response. A review in the theme: cellular responses to hypoxia. Am J Physiol Cell Physiol 2015; 309: C569–79. 26310815 10.1152/ajpcell.00207.2015 PMC4628936

[114] Burke B , Giannoudis A , Corke KP *et al*. Hypoxia‐induced gene expression in human macrophages: implications for ischemic tissues and hypoxia‐regulated gene therapy. Am J Clin Pathol 2003; 163: 1233–43. 10.1016/S0002-9440(10)63483-9 PMC1868302 14507633

[115] Ruf M , Moch H , Schraml P . PD‐L1 expression is regulated by hypoxia inducible factor in clear cell renal cell carcinoma. Int J Cancer 2016; 139: 396–403. 26945902 10.1002/ijc.30077

[116] Semaan A , Dietrich D , Bergheim D *et al*. CXCL12 expression and PD‐L1 expression serve as prognostic biomarkers in HCC and are induced by hypoxia. Virchows Arch 2016; 470: 185–96. 27913861 10.1007/s00428-016-2051-5

[117] Messai Y , Gad S , Noman MZ *et al*. Renal cell carcinoma programmed death‐ligand 1, a new direct target of hypoxia‐inducible factor‐2 alpha, is regulated by von Hippel–Lindau gene mutation status. Eur Urol 2015; 70: 623–32. 26707870 10.1016/j.eururo.2015.11.029

[118] Noman MZ , Desantis G , Janji B *et al*. PD‐L1 is a novel direct target of HIF‐1α, and its blockade under hypoxia enhanced: MDSC‐mediated T cell activation. J Exp Med 2014; 211: 781–90. 24778419 10.1084/jem.20131916 PMC4010891

[119] Deng J , Li J , Sarde A *et al*. Hypoxia‐induced VISTA promotes the suppressive function of myeloid‐derived suppressor cells in the tumor microenvironment. Cancer Immunol Res 2019; 7: 1079–90. 31088847 10.1158/2326-6066.CIR-18-0507 PMC6606337

[120] Zhang H , Lu H , Xiang L *et al*. HIF‐1 regulates CD47 expression in breast cancer cells to promote evasion of phagocytosis and maintenance of cancer stem cells. Proc Natl Acad Sci USA 2015; 112: E6215–23. 26512116 10.1073/pnas.1520032112 PMC4653179

[121] Logtenberg MEW , Scheeren FA , Schumacher TN . The CD47‐SIRPα immune checkpoint. Immunity 2020; 52: 742–52. 32433947 10.1016/j.immuni.2020.04.011 PMC7340539

[122] Barsoum IB , Hamilton TK , Li X *et al*. Hypoxia induces escape from innate immunity in cancer cells via increased expression of ADAM10: role of nitric oxide. Cancer Res 2011; 71: 7433–41. 22006996 10.1158/0008-5472.CAN-11-2104

[123] Siemens DR , Hu N , Sheikhi AK *et al*. Hypoxia increases tumor cell shedding of MHC class I chain‐related molecule: role of nitric oxide. Cancer Res 2008; 68: 4746–53. 18559521 10.1158/0008-5472.CAN-08-0054

[124] Baginska J , Viry E , Berchem G *et al*. Granzyme B degradation by autophagy decreases tumor cell susceptibility to natural killer‐mediated lysis under hypoxia. Proc Natl Acad Sci USA 2013; 110: 17450–5. 24101526 10.1073/pnas.1304790110 PMC3808626

[125] Viry E , Baginska J , Berchem G *et al*. Autophagic degradation of GZMB/granzyme B: a new mechanism of hypoxic tumor cell escape from natural killer cell‐mediated lysis. Autophagy 2014; 10: 173–5. 24248158 10.4161/auto.26924 PMC4389872

[126] Overgaard J , Sand Hansen H , Overgaard M *et al*. A randomized double‐blind phase III study of nimorazole as a hypoxic radiosensitizer of primary radiotherapy in supraglottic larynx and pharynx carcinoma. Results of the danish head and neck cancer study (DAHANCA) protocol 5‐85. Radiother Oncol 1998; 46: 135–46. 9510041 10.1016/s0167-8140(97)00220-x

[127] Grogan M , Thomas GM , Melamed I *et al*. The importance of hemoglobin levels during radiotherapy for carcinoma of the cervix. Cancer 1999; 86: 1528–36. 10526282 10.1002/(sici)1097-0142(19991015)86:8<1528::aid-cncr20>3.0.co;2-e

[128] Dusault LA . The effect of oxygen on the response of spontaneous tumours in mice to radiotherapy. Br J Radiol 1963; 36: 749–54. 14067659 10.1259/0007-1285-36-430-749

[129] Aquino‐Parsons C , Hukin J , Green A . Concurrent carbogen and radiation therapy in children with high‐risk brainstem gliomas. Pediatr Blood Cancer 2008; 41: 397–9. 10.1002/pbc.21057 17009221

[130] Hoskin PJ , Rojas AM , Bentzen SM , Saunders MI . Radiotherapy with concurrent carbogen and nicotinamide in bladder carcinoma. J Clin Oncol 2010; 28: 4912–8. 20956620 10.1200/JCO.2010.28.4950

[131] Janssens GORJ , Rademakers SE , Terhaard CHJ *et al*. Accelerated radiotherapy with carbogen and nicotinamide for laryngeal cancer: results of a phase III randomized trial. J Clin Oncol 2012; 30: 1777–83. 22508814 10.1200/JCO.2011.35.9315

[132] Bussink J , Kaanders JHAM , Strik A , van der Kogel AJ . Effects of nicotinamide and carbogen on oxygenation in human tumor xenografts measured with luminescence based fiber‐optic probes. Radiother Oncol 2000; 57: 21–30. 11033185 10.1016/s0167-8140(00)00275-9

[133] Hunter FW , Wouters BG , Wilson WR . Hypoxia‐activated prodrugs: paths forward in the era of personalised medicine. Br J Cancer 2016; 114: 1071–7. 27070712 10.1038/bjc.2016.79 PMC4865974

[134] Jayaprakash P , Ai M , Liu A *et al*. Targeted hypoxia reduction restores T cell infiltration and sensitizes prostate cancer to immunotherapy. J Clin Invest 2018; 128: 5137–49. 30188869 10.1172/JCI96268 PMC6205399

[135] Peeters SGJA , Zegers CML , Biemans R *et al*. TH‐302 in combination with radiotherapy enhances the therapeutic outcome and is associated with pretreatment [F‐18]HX4 hypoxia PET imaging. Clin Cancer Res 2015; 21: 2984–92. 25805800 10.1158/1078-0432.CCR-15-0018

[136] Hajj C , Russell J , Hart CP *et al*. A combination of radiation and the hypoxia‐activated prodrug evofosfamide (TH‐302) is efficacious against a human orthotopic pancreatic tumor model. Transl Oncol 2017; 10: 760–5. 28778024 10.1016/j.tranon.2017.06.010 PMC5538966

[137] Takakusagi Y , Kishimoto S , Naz S *et al*. Radiotherapy synergizes with the hypoxia‐activated prodrug evofosfamide: in vitro and in vivo studies. Antioxid Redox Signal 2018; 28: 131–40. 28741367 10.1089/ars.2017.7106 PMC5725636

[138] Li Y , Zhao L , Li X‐F . The hypoxia‐activated prodrug TH‐302: exploiting hypoxia in cancer therapy. Front Pharmacol 2021; 12: 636892. 33953675 10.3389/fphar.2021.636892 PMC8091515

[139] Secomb TW , Hsu R , Ong ET , Gross JF , Dewhirst MW . Analysis of the effects of oxygen supply and demand on hypoxic fraction in tumors. Acta Oncol 1995; 34: 313–6. 7779415 10.3109/02841869509093981

[140] Ashton TM , Gillies McKenna W , Kunz‐Schughart LA , Higgins GS . Oxidative phosphorylation as an emerging target in cancer therapy. Clin Cancer Res 2018; 24: 2482–90. 29420223 10.1158/1078-0432.CCR-17-3070

[141] Gopal YNV , Gammon S , Prasad R *et al*. A novel mitochondrial inhibitor blocks MAPK pathway and overcomes MAPK inhibitor resistance in melanoma. Clin Cancer Res 2019; 25: 6429–42. 31439581 10.1158/1078-0432.CCR-19-0836 PMC6825560

[142] Birkenmeier K , Se S , Wittig I *et al*. Hodgkin and Reed‐Sternberg cells of classical Hodgkin lymphoma are highly dependent on oxidative phosphorylation. Int J Cancer 2016; 138: 2231–46. 26595876 10.1002/ijc.29934

[143] Whitaker‐Menezes D , Martinez‐Outschoorn UE , Flomenberg N *et al*. Hyperactivation of oxidative mitochondrial metabolism in epithelial cancer cells in situ: visualizing the therapeutic effects of metformin in tumor tissue. Cell Cycle 2011; 10: 4047–64. 22134189 10.4161/cc.10.23.18151 PMC3272287

[144] Viale A , Pettazzoni P , Lyssiotis CA *et al*. Oncogene ablation‐resistant pancreatic cancer cells depend on mitochondrial function. Nature 2014; 514: 628–32. 25119024 10.1038/nature13611 PMC4376130

[145] Zannella VE , Dal Pra A , Muaddi H *et al*. Reprogramming metabolism with metformin improves tumor oxygenation and radiotherapy response. Clin Cancer Res 2013; 19: 6741–50. 24141625 10.1158/1078-0432.CCR-13-1787

[146] Shen H , Yu M , Tsoli M *et al*. Targeting reduced mitochondrial DNA quantity as a therapeutic approach in pediatric high‐grade gliomas. J Neurooncol 2020; 22: 139–51. 10.1093/neuonc/noz140 PMC6954438 31398252

[147] Chevalier B , Pasquier D , Lartigau EF *et al*. Metformin: (future) best friend of the radiation oncologist? Radiother Oncol 2020; 151: 95–105. 32592892 10.1016/j.radonc.2020.06.030

[148] Scharping NE , Menk AV , Whetstone RD , Zeng X , Delgoffe GM . Efficacy of PD‐1 blockade is potentiated by metformin‐induced reduction of tumor hypoxia. Cancer Immunol Res 2017; 5: 9–16. 27941003 10.1158/2326-6066.CIR-16-0103 PMC5340074

[149] Ashton TM , Fokas E , Kunz‐Schughart LA *et al*. The anti‐malarial atovaquone increases radiosensitivity by alleviating tumour hypoxia. Nat Commun 2016; 7: 12308. 27453292 10.1038/ncomms12308 PMC4962491

[150] Wang S , Zhou X , Zeng Z *et al*. Atovaquone‐HSA nano‐drugs enhance the efficacy of PD‐1 blockade immunotherapy by alleviating hypoxic tumor microenvironment. J Nanobiotechnology 2021; 19: 302. 34600560 10.1186/s12951-021-01034-9 PMC8487475

[151] Skwarski M , McGowan DR , Belcher E *et al*. Mitochondrial inhibitor atovaquone increases tumor oxygenation and inhibits hypoxic gene expression in patients with non‐small cell lung cancer. Clin Cancer Res 2021; 27: 2459–69. 33597271 10.1158/1078-0432.CCR-20-4128 PMC7611473

[152] Benej M , Hong X , Vibhute S *et al*. Papaverine and its derivatives radiosensitize solid tumors by inhibiting mitochondrial metabolism. Proc Natl Acad Sci USA 2018; 115: 10756–61. 30201710 10.1073/pnas.1808945115 PMC6196495

[153] Najjar YG , Menk AV , Sander C *et al*. Tumor cell oxidative metabolism as a barrier to PD‐1 blockade immunotherapy in melanoma. JCI Insight 2019; 4: e124989. 10.1172/jci.insight.124989 PMC6483505 30721155

[154] Ho P‐C , Bihuniak Jessica D , Macintyre Andrew N *et al*. Phosphoenolpyruvate is a metabolic checkpoint of anti‐tumor T cell responses. Cell 2015; 162: 1217–28. 26321681 10.1016/j.cell.2015.08.012 PMC4567953

[155] Hugo W , Zaretsky JM , Sun L *et al*. Genomic and transcriptomic features of response to anti‐PD‐1 therapy in metastatic melanoma. Cell 2016; 165: 35–44. 26997480 10.1016/j.cell.2016.02.065 PMC4808437

[156] Chen D , Barsoumian HB , Fischer G *et al*. Combination treatment with radiotherapy and a novel oxidative phosphorylation inhibitor overcomes PD‐1 resistance and enhances antitumor immunity. J Immunother Cancer 2020; 8: e000289. 32581056 10.1136/jitc-2019-000289 PMC7319777

[157] Gallez B , Neveu M‐A , Danhier P , Jordan BF . Manipulation of tumor oxygenation and radiosensitivity through modification of cell respiration. A critical review of approaches and imaging biomarkers for therapeutic guidance. Biochim Biophys Acta Bioenerg 2017; 1858: 700–11. 28088332 10.1016/j.bbabio.2017.01.002

[158] Jonasch E , Donskov F , Iliopoulos O *et al*. Belzutifan for renal cell carcinoma in von Hippel–Lindau disease. N Engl J Med 2021; 385: 2036–46. 34818478 10.1056/NEJMoa2103425 PMC9275515

[159] Bhattarai D , Xu X , Lee K . Hypoxia‐inducible factor‐1 (HIF‐1) inhibitors from the last decade (2007 to 2016): a “structure–activity relationship” perspective. Med Res Rev 2018; 38: 1404–42. 29278273 10.1002/med.21477

[160] Fallah J , Rini BI . HIF inhibitors: status of current clinical development. Curr Oncol Rep 2019; 21: 1–10. 10.1007/s11912-019-0752-z 30671662

[161] Luo F , Lu F‐T , Cao J‐X *et al*. HIF‐1α inhibition promotes the efficacy of immune checkpoint blockade in the treatment of non‐small cell lung cancer. Cancer Lett 2022; 531: 39–56. 35090965 10.1016/j.canlet.2022.01.027

[162] Lequeux A , Noman MZ , Xiao M *et al*. Targeting HIF‐1 alpha transcriptional activity drives cytotoxic immune effector cells into melanoma and improves combination immunotherapy. Oncogene 2021; 40: 4725–35. 34155342 10.1038/s41388-021-01846-x PMC8282500

[163] Palayoor ST , Mitchell JB , Cerna D , DeGraff W , John‐Aryankalayil M , Coleman CN . PX‐478, an inhibitor of hypoxia‐inducible factor‐1α, enhances radiosensitivity of prostate carcinoma cells. Int J Cancer 2008; 123: 2430–7. 18729192 10.1002/ijc.23807 PMC4277812

[164] Cui H , Qin Q , Yang M *et al*. Bortezomib enhances the radiosensitivity of hypoxic cervical cancer cells by inhibiting HIF‐1α expression. Int J Clin Exp Pathol 2015; 8: 9032–41. 26464645 PMC4583877

[165] Wang D , Qin Q , Jiang Q‐J , Wang D‐F . Bortezomib sensitizes esophageal squamous cancer cells to radiotherapy by suppressing the expression of HIF‐1α and apoptosis proteins. J Xray Sci Technol 2016; 24: 639–46. 27080362 10.3233/XST-160571

[166] Staab A , Loeffler J , Said HM *et al*. Effects of HIF‐1 inhibition by chetomin on hypoxia‐related transcription and radiosensitivity in HT 1080 human fibrosarcoma cells. BMC Cancer 2007; 7: 213. 17999771 10.1186/1471-2407-7-213 PMC2200672

[167] Kessler J , Hahnel A , Wichmann H *et al*. HIF‐1α inhibition by siRNA or chetomin in human malignant glioma cells: effects on hypoxic radioresistance and monitoring via CA9 expression. BMC Cancer 2010; 10: 605. 21050481 10.1186/1471-2407-10-605 PMC2992520

